# PolyReco: A Method to Automatically Label Collinear Regions and Recognize Polyploidy Events Based on the *K*_*S*_ Dotplot

**DOI:** 10.3389/fgene.2022.842387

**Published:** 2022-04-20

**Authors:** Fushun Wang, Kang Zhang, Ruolan Zhang, Hongquan Liu, Weijin Zhang, Zhanxiao Jia, Chunyang Wang

**Affiliations:** ^1^ Department of Information Science and Technology, Hebei Agricultural University, Baoding, China; ^2^ Hebei Key Laboratory of Agricultural Big Data, Baoding, China; ^3^ Department of Life Science, Hebei Agricultural University, Baoding, China; ^4^ State Key Laboratory of North China Crop Improvement and Regulation, Hebei Agricultural University, Baoding, China; ^5^ Hebei Key Laboratory of Plant Physiology and Molecular Pathology, Hebei Agricultural University, Baoding, China; ^6^ Department of Urban and Rural Construction, Hebei Agricultural University, Baoding, China

**Keywords:** clustering, collinearity fragment, polyploidy, DBSCAN, chromosome

## Abstract

Polyploidization plays a critical role in producing new gene functions and promoting species evolution. Effective identification of polyploid types can be helpful in exploring the evolutionary mechanism. However, current methods for detecting polyploid types have some major limitations, such as being time-consuming and strong subjectivity, etc. In order to objectively and scientifically recognize collinearity fragments and polyploid types, we developed PolyReco method, which can automatically label collinear regions and recognize polyploidy events based on the *K*_*S*_ dotplot. Combining with whole-genome collinearity analysis, PolyReco uses DBSCAN clustering method to cluster *K*_*S*_ dots. According to the distance information in the *x*-axis and *y*-axis directions between the categories, the clustering results are merged based on certain rules to obtain the collinear regions, automatically recognize and label collinear fragments. According to the information of the labeled collinear regions on the *y*-axis, the polyploidization recognition algorithm is used to exhaustively combine and obtain the genetic collinearity evaluation index of each combination, and then draw the genetic collinearity evaluation index graph. Based on the inflection point on the graph, polyploid types and related chromosomes with polyploidy signal can be detected. The validation experiments showed that the conclusions of PolyReco were consistent with the previous study, which verified the effectiveness of this method. It is expected that this approach can become a reference architecture for other polyploid types classification methods.

## Introduction

Studying the process of polyploidization is essential for the in-depth understanding of evolutionary laws (Marcet-Houben and Gabaldón 2015), and exploring the stability and chromosome rearrangement of the genome. Polyploidization of gymnosperms and almost all angiosperms are considered to be the main reason for the diversity of land plants (Li et al., 2016; Hao et al., 2017). Polyploidy can produce a large number of duplicated genes in the genome (Wang et al., 2018). These genes may play an important role in functional evolution, environmental adaptation, and new species formation (Wang et al., 2017; Wang et al., 2019). The recombination of some homologous chromosomes after polyploidization often causes the instability of the genome structure, and processes such as chromosome breakage and fusion often occur, which can lead to large-scale duplicated gene loss in the genome (Wang et al., 2007; Wang et al., 2011). If two species have a common ancestor, after polyploidization, although there will be differences between the genomes, the two species still have a relatively close relationship. This close relationship can be expressed in the form of collinearity. The more complete the collinearity fragment, the closer relationship between the two species is. Exploring the collinearity between species has big significance in understanding the origin of species and the evolution of the genome.

Cheng et al. (2019) drew a *K*_*S*_ dotplot of homologous genes within Spirogloea muscicola, and found that it had recently experienced a whole genome triplication event. Through collinearity analysis, Wang et al., 2011 found that *Brassica rapa* and *Arabidopsis thaliana* experienced a whole genome triplication event. By constructing a phylogenetic gene tree, Dong et al. (2021) revealed that a whole-genome duplication event occurred in Magnoliales and Laurales. Xu et al. (2020) by drawing a phylogenetic gene tree, found that Scutellaria baicalensis and Scutellaria barbata had a whole-genome duplication event about 13.28 million years ago. Yan et al., 2021 by drawing the distribution graph of the synonymous substitution rate (*K*_*S*_), found two WGD events in Juglans mandshurica and Juglans regia. By drawing the distribution graph of the synonymous substitution rate (*K*_*S*_).

Although the *K*_*S*_ distribution graph combined with the molecular clock (Miyata et al., 1980) can calculate the doubling time, it is a challenge to determine the collinearity information between the chromosomes. In addition, the above method, which injects prior knowledge, manually marks the collinear area by observing the atlas, and then recognizes the polyploidization through the combination of the regions. This kind of recognition method has low recognition efficiency, high dependence on prior knowledge, as well as strong subjectivity, and easy to introduce human error. Due to the lack of objective evaluation criteria, the identification of the polyploid types of is still very challenging. In terms of the types of polyploidization and the choice of chromosomes, the same atlas will cause different personal perceptions. This deviation will affect the subsequent research on chromosome rearrangement (Zhang et al., 2021). Therefore, we develop a computational model PolyReco to accurately identify and characterize some polyploid types in atlas.

Considering only *K*_*S*_ values for identifying polyploid types might be insufficient, we add the gene positional information in PolyReco. Genes are aligned in sequential order on each of the chromosomes, so incorporating the gene positional information on chromosomes with *K*_*S*_ values will likely increase the accuracy of polyploid type classification. In this study, sequence comparisons were performed based on the whole genome data of *Vitis vinifera* and *Salix sinopurpurea*, combined with whole genome collinearity analysis, to obtain the summary data of homology information and *K*_*S*_ values between genomes. PolyReco comprehensively utilizes digital image processing technology and DBSCAN method, and realizes the automatic recognition and labeling of the collinear region based on the *K*_*S*_ dotplot of homologous genes. The model uses the collinear area as the unit and combines the combination strategy to construct the combination evaluation standard. According to the performance of the chromosome combination, determine the specific polyploidization and draw the combined figure of the polyploidization. This study aims to develop a polyploidization classification tool, which has the potential to take chromosome position information into account with the *K*_*S*_ values for boosting polyploid type prediction performance.

## Materials and Methods

### Data Sources

With the whole genome data of *Salix sinopurpurea* (Spu) and *Brassica rapa* (Bra) as the main research materials, comparative genomics was used to compare the collinearity between *Salix sinopurpurea* and the reference genome *Vitis vinifera* (Vvi), *Brassica rapa* and the reference genome *Arabidopsis thaliana* (Ath).

Genomes and their gene annotations of *Salix sinopurpurea* and *Vitis vinifera* were downloaded from Joint Genome Institute. Download the required documents for *Brassica rapa* and *Arabidopsis thaliana* at http://brassicadb.org/and http://www.arabidopsis.org/shangxia, respectively.

### Preprocessing of the Data Sources

Due to the huge amount of original genome data, in order to extract target data from the genome sequence and annotation files, the downloaded genome data is processed with a custom python script to obtain the blast results, which is convenient for subsequent research and analysis. Screen the original data of species genomes, information was extracted from the genome annotation files, which include chromosome number, gene start and end positions, gene transcription direction, and gene ID information, and then rename the gene ID and the number of the genes was given in order of their appearance on chromosomes. Map the gene ID in the CDS sequence and protein sequence file to the new ID of the corresponding gene in the genome annotation file. Label the processed genomic data with a unified naming method.

### Homologous Sequence Alignment

Blastp was used to explore to align genomic sequences of different species. Screen out gene pairs with the expected value (E-value) not greater than 10–5 and score evaluation (Score) higher than 100, so that the subsequent genome collinearity analysis results are more reliable.

### Draw the *K*_*S*_ Dotplot of Homologous Genes

The WGDI (Sun et al., 2021) use MAFFT (Wong, Suchard, and Huelsenbeck 2008) or MUSCLE (Edgar 2004) to perform multiple sequence alignment, and calculates the synonymous substitution rate using the yn00 (Yang et al., 2000) or ng86 (Nei and Gojobori 1986) program of the PAML package. Finally, the visualization is realized by extracting block, and then output blockinfo file.

### Collinear Fragment Labeling Method Based on Clustering

In this paper, the input for DBSCAN requires the blockinfo file generated by WGDI and the chromosome length information (len file) of the two species. By setting the epsilon (eps) and minimum points (MinPts), cluster analysis is performed on the collinearity fragments in the *K*_*S*_ dotplot. The collinear region was then obtained from the clustering results combined with certain rules for merging. And then realizes the automatic identification and labeling of the collinear region. The comparison result of a chromosome of the target species and a chromosome of the reference species is shown as a cell on the *K*_*S*_ dotplot, that is, a comparison unit.

**FIGURE 1:**
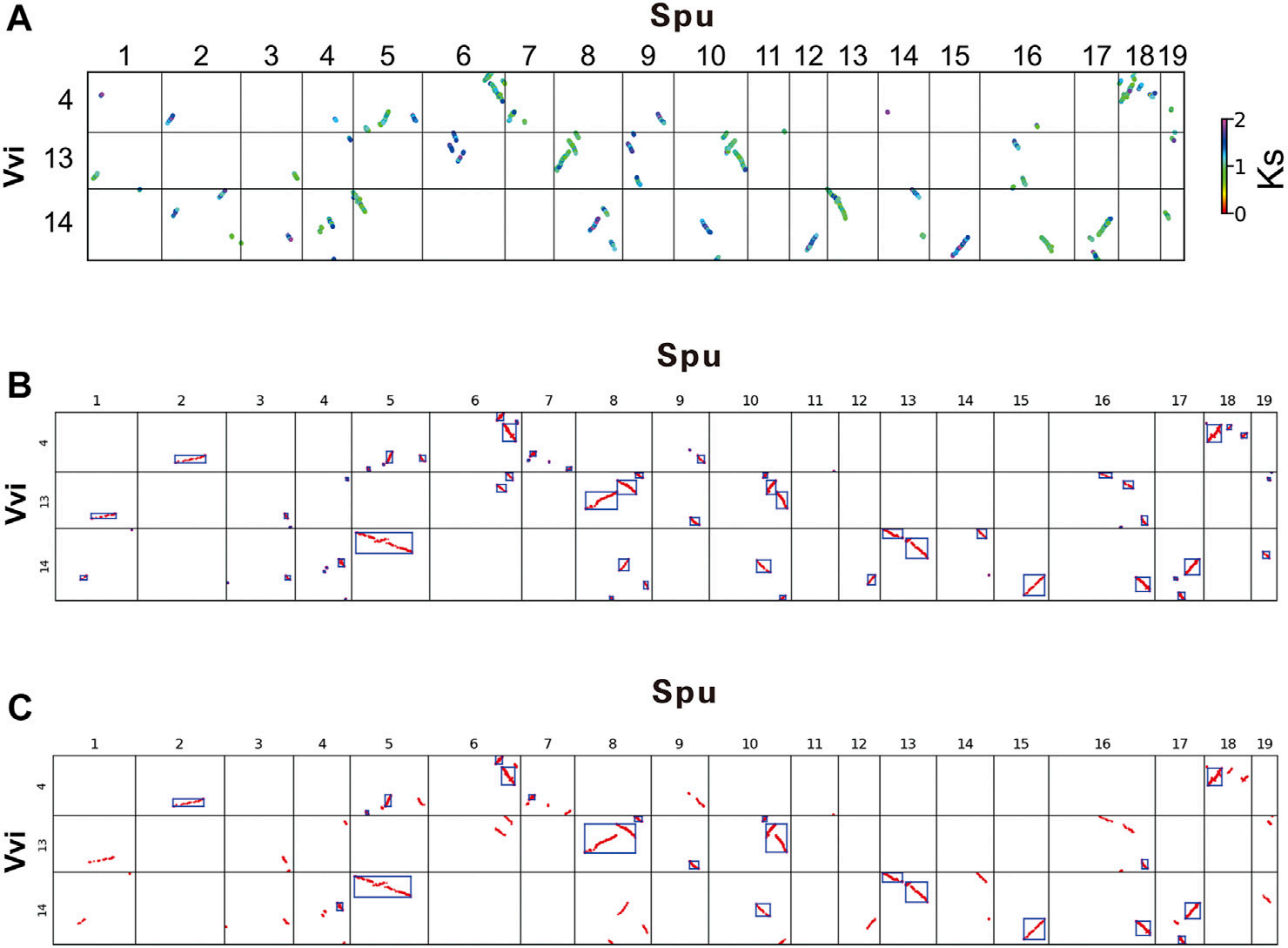
**(A)** *K*_*S*_ dotplot between Salix sinopurpurea and Vitis vinifera genome homologous genes **(B)** DBSCAN cluster recognition effect figure **(C)** Automatic label result of collinearity fragments based on DBSCAN.

The *K*_*S*_ dotplot between *Salix sinopurpurea* and *Vitis vinifera* genome homologous genes drawn by wgdi (Figure 1A). The horizontal axis represents the chromosome of the target species (*Salix sinopurpurea*) and the vertical represents the chromosome of the reference species (*Vitis vinifera*). On the *K*_*S*_ dotplot, the chromosome number of *Salix sinopurpurea* is shown from left to right, and the chromosome number of *Vitis vinifera* is shown from top to bottom. The *K*_*S*_ value ranges from 0.00 to 2.00. As shown in the figure, different colored points correspond to different *K*_*S*_ values. It can be observed in Figure 1A, in addition to the clear and complete homologous fragments of grape chromosome 4 with *Salix sinopurpurea* chromosomes 6 and 18, it also has fuzzy and unclear homologous fragments with *Salix sinopurpurea* chromosomes 1, 2 and 4. The reason why these fragments are unclear and incomplete is that they are doubled by the whole genome triplication events shared by older dicots. The collinearity of the homologous fragments produced by the whole genome triplication events shared by ancient dicotyledons is far inferior to that of the whole genome duplication events shared by the Salicaceae. The specific manifestation is that the *K*_*S*_ value is significantly large, belongs to the blue-purple system, scarce and fragmented seriously. The results showed the structural similarities and differences between genomes. The generated data and pictures provide references for follow-up research.

Using the DBSCAN algorithm, by setting the eps to 50 and the MinPts to 3, cluster the *K*_*S*_ dotplot between *Salix sinopurpurea* and *Vitis vinifera* genome homologous genes. The algorithm outputs the clustering result figure (Figure 1B), in which each category is represented by a rectangular box. The DBSCAN can cluster out complete collinearity fragments, in grape chromosome 14 and *Salix sinopurpurea* chromosome 5, as well as in grape chromosome 14 and *Salix sinopurpurea* chromosome 15. It also can identify fragmented collinearity fragments in grape chromosome 4 and *Salix sinopurpurea* chromosome 5, grape chromosome 13 and *Salix sinopurpurea* chromosome 16. These will accurately reflect the relationship between the collinearity fragments and improve the subsequent combination effect.

The model sorts the category in the same comparison unit from top to bottom to generate ID, and calculates the number of homologous gene points in the box (num), the length of the box (*y*1-*y*2), and the width of the box (*x*1-*x*2). The model then generates cluster.csv files that contain the target species chromosome number (chr1), the reference species chromosome number (chr2), ID, and the horizontal and vertical coordinates of the upper left corner point are l_*x* and l_*y*, respectively, num, the horizontal and vertical coordinates of the lower right point are r_*x* and r_*y*, respectively, *y*1-*y*2, and *x*1-*x*2. Part of the data in the cluster.csv file of grape chromosome 13 is shown in Table 1. Among them, chromosome 13 and chromosome 1 form a class. The coordinates of the upper left corner of this class are 145, 354, and the coordinates of the lower right corner are 257, 228. The number of homologous genes contained is 32. The length of the cluster box is 126 coordinate lengths, and the width is 112 coordinate lengths.

**TABLE 1:** Partial data of grape chromosome 13 cluster.csv file.

chr1	chr2	id	l_x	l_y	num	r_x	r_y	y1-y2	x1-x2
1	13	1	145	354	32	257	228	126	112
4	13	1	1,266	1,153	10	1,310	1,097	56	44
6	13	1	838	1,267	17	913	1,097	170	75
6	13	2	735	994	26	834	840	154	99
8	13	1	592	1,267	56	671	1,155	112	79

In order to perform a combined analysis on the identified collinearity fragments, the model read the cluster.csv file generated by clustering. The model uses *y*_gap and *x*_gap, which represents the gap in the longitudinal and horizontal directions of adjacent collinear segments, as the basis for judging overlap. The location information of the gene is combined to set the parameters gap and Slen. In the comparison unit, the parameter gap represents the mean value of *y*_gap. Through a series of experiments and continuous optimization of parameter selection, it is finally determined that 1/6 of the corresponding chromosome length of the target species is the value of parameter Slen. For all collinearity fragments whose num is greater than the specified value, one condition is that the collinearity fragments do not overlap, another is overlap. In the first condition, collinear fragments will be merged if 0 ≤*y*_gap ≤ gap and 0 ≤*x*_gap ≤ Slen. And in the second condition, there is overlap in the *y*-axis direction, they will be merged when it meets 0 ≤ *x*_gap < Slen; if there is overlap in the *x*-axis direction, when it meets 0 ≤ *y*_gap ≤ gap, merge them. Finally, the model output the clustering result graph (Figure 1C), in which the merged result is marked with a rectangular box, and the combine.csv file is generated.

The content of the combine.csv file is the same as the cluster.csv file. Table 2 shows some data of the combine.csv file. Among them, chromosome 13 and chromosome 8 form two classes. The coordinates of the upper left corner of the first class are 592, 1,264, and the lower right corner are 671, 1,155. The number of homologous genes contained in this class is 56. The length of the labeled box ∆y is 112 coordinate length, and the width ∆x is 79 coordinate length; the upper left corner coordinate of the second class are 78, 1,089, and the lower right corner are 605, 446. The number of homologous genes contained in this class is 239, ∆y is 643 coordinate length, ∆x is 537.

**TABLE 2:** Partial data of grape chromosome 13 combine.csv file.

chr1	chr2	id	l_x	l_y	num	r_x	r_y	∆y	∆x
8	13	1	592	1,267	56	671	1,155	112	79
8	13	2	78	1,089	239	605	446	643	527
9	13	1	378	257	63	478	89	168	100
10	13	1	1,327	1,267	65	1,426	1,157	110	99
10	13	2	1,419	1,088	276	1,934	457	631	515

### Polyploidy Recognition Algorithm

In order to determine the polyploid types and related chromosomes of the species, we develop the polyploidy recognition algorithm. The algorithm read the generated combine.csv file, look for the labeled box with the largest ∆y, mark it, and then look up and down to find the labeled box with the length less than it. The result.csv file will be built by adopting exhaustive above process. Among them, comro represents the combination round, sumy represents the sum of ∆y in same combination round. In order to determine the specific polyploidy of the species, the gene collinearity evaluation index line chart is drawn. The horizontal is the combined round, and the vertical is the corresponding gene collinearity evaluation index. The significant inflection point in the line chart represents the corresponding polyploid type. The gene collinearity evaluation index (MI) was calculated by dividing the cumulative collinearity fragments length to the corresponding chromosome length of the reference species in the len file to describe the performance of the polyploidy in the corresponding combination round, and the larger its value, the better the performance.

$$MI=\frac{\sum_{m=1}^{n}\Delta y_{m}}{len_{i}}$$

Where $\Delta y_{m}$ and *n* are the length and number of collinearity fragments in the same combination round, respectively; $\;len_{i}$ is the corresponding chromosome length of the reference species, *i* is the corresponding chromosome number.

After determining the combination round, output the combined result graph. To describe the analysis of input, output and fetching the final results of the analysis, we made pseudo code. The pseudo code of the chromosome collinearity fragment labeling and polyploidy recognition algorithm is shown as below for a better understanding of the context and better assess the relevance of this paper.

Algorithm 1 Chromosome collinearity fragment labeling and polyploidy recognition algorithm.

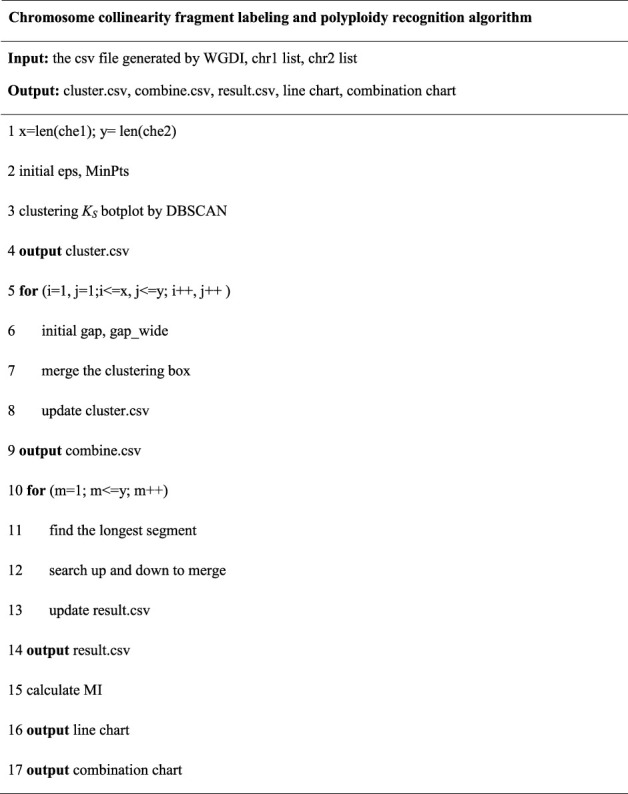

## Results

### *Salix sinopurpurea* Polyploidy Recognition

**FIGURE 2:**
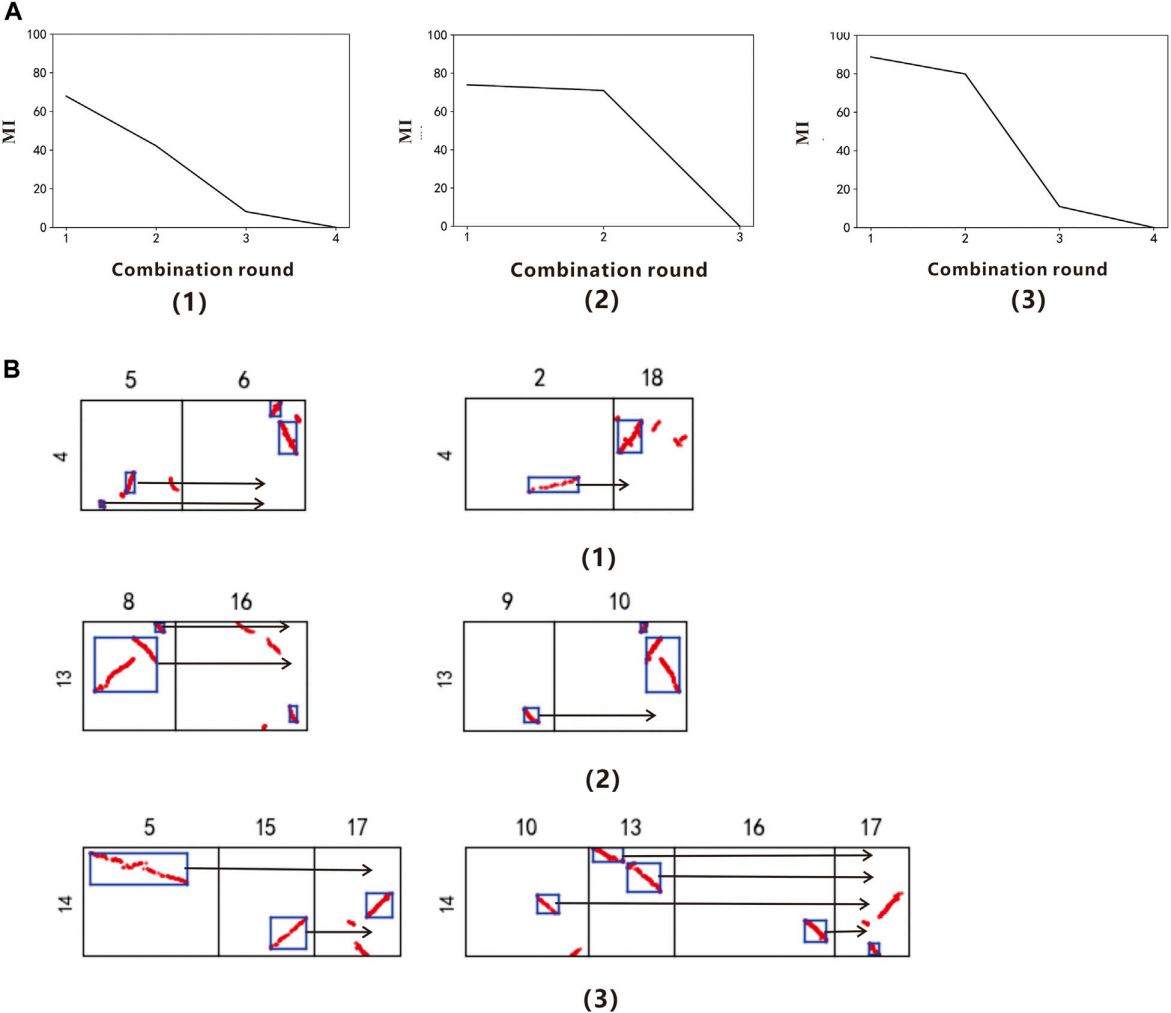
**(A)** Collinearity evaluation index line chart of Vitis vinifera Chr.4, Chr.13, and Chr.14 **(B)** Combination figure of Salix sinopurpurea polyploidy. **(A,B)** correspond to each other. (1) The grape chromosome 4 (2) The grape chromosome 13 (3) The grape chromosome 14.

Using PolyReco to objectively determine the polyploid types of *Salix sinopurpurea*. The model use *Vitis vinifera* as the reference genome to identify the target species *Salix sinopurpurea* polyploid type, and read the data of *Vitis vinifera* chromosome 4, 13 and 14. The DBSCAN algorithm obtains the collinearity fragments, and get the combine.csv file. Then using the polyploidy recognition algorithm to combine the labeled boxes exhaustively, and get the gene collinearity evaluation index table of each chromosome in different combination rounds (Supplementary Table S1). In the colinearity evaluation index line chart (Figure 2A), we can find that chromosomes 4, 13, and 14 of *Vitis vinifera* have obvious inflection points when the combined round is 2. Therefore, it is determined that the *Salix sinopurpurea* has a whole genome duplication event recently. This conclusion can be found in Wang et al., 2011.

After determining the specific polyploidy, we can output the information of the labeled box participating in the polyploidy, and obtain the result.csv file of *Vitis vinifera* chromosomes 4, 13, and 14, in which the data of chromosome 13 is shown in Table 3.

**TABLE 3:** *Vitis vinifera* Chr. 13 result.csv file.

chr1	chr2	id	l_x	l_y	num	r_x	r_y	Sumy	Δy	Δx	Comro
8	13	2	78	1,089	239	605	446	947	643	527	1
8	13	1	592	1,267	56	671	1,155	947	112	79	1
16	13	1	1,163	283	42	1,235	91	947	192	72	1
10	13	2	1,419	1,088	276	1,934	457	909	631	515	2
10	13	1	1,327	1,267	65	1,426	1,157	909	110	99	2
9	13	1	378	257	63	478	89	909	168	100	2

According to the result.csv file, output the combined figure of the *Salix sinopurpurea* polyploidy (Figure 2B). When the combination round is 2, get the two groups with the highest scores, among which the chromosomes 5 and 6 of the *Salix sinopurpurea* can be combined into a relatively complete chromosome 4 of *Vitis vinifera*, the corresponding MI is 67.87%; the chromosomes 2 and 18 of the *Salix sinopurpurea* can be combined into a relatively complete chromosome 4 of *Vitis vinifera*, and the corresponding MI is 42.19%. The chromosomes 8 and 16 of the *Salix sinopurpurea* can be combined into a relatively complete *Vitis vinifera* chromosome 13 with MI of 73.93%; the chromosomes 9 and 10 of the *Salix sinopurpurea* can be combined into a relatively complete *Vitis vinifera* chromosome 4, MI is 70.96%. The chromosomes 5, 15, and 17 of *Salix sinopurpurea* can be combined into a relatively complete *Vitis vinifera* chromosome 14 with MI of 88.74%; the chromosomes 10, 13, 16, and 17 of *Salix sinopurpurea* can be combined into a relatively complete *Vitis vinifera* chromosome 14, MI is 79.88%.

### *Brassica rapa* Polyploidy Recognition

**FIGURE 3:**
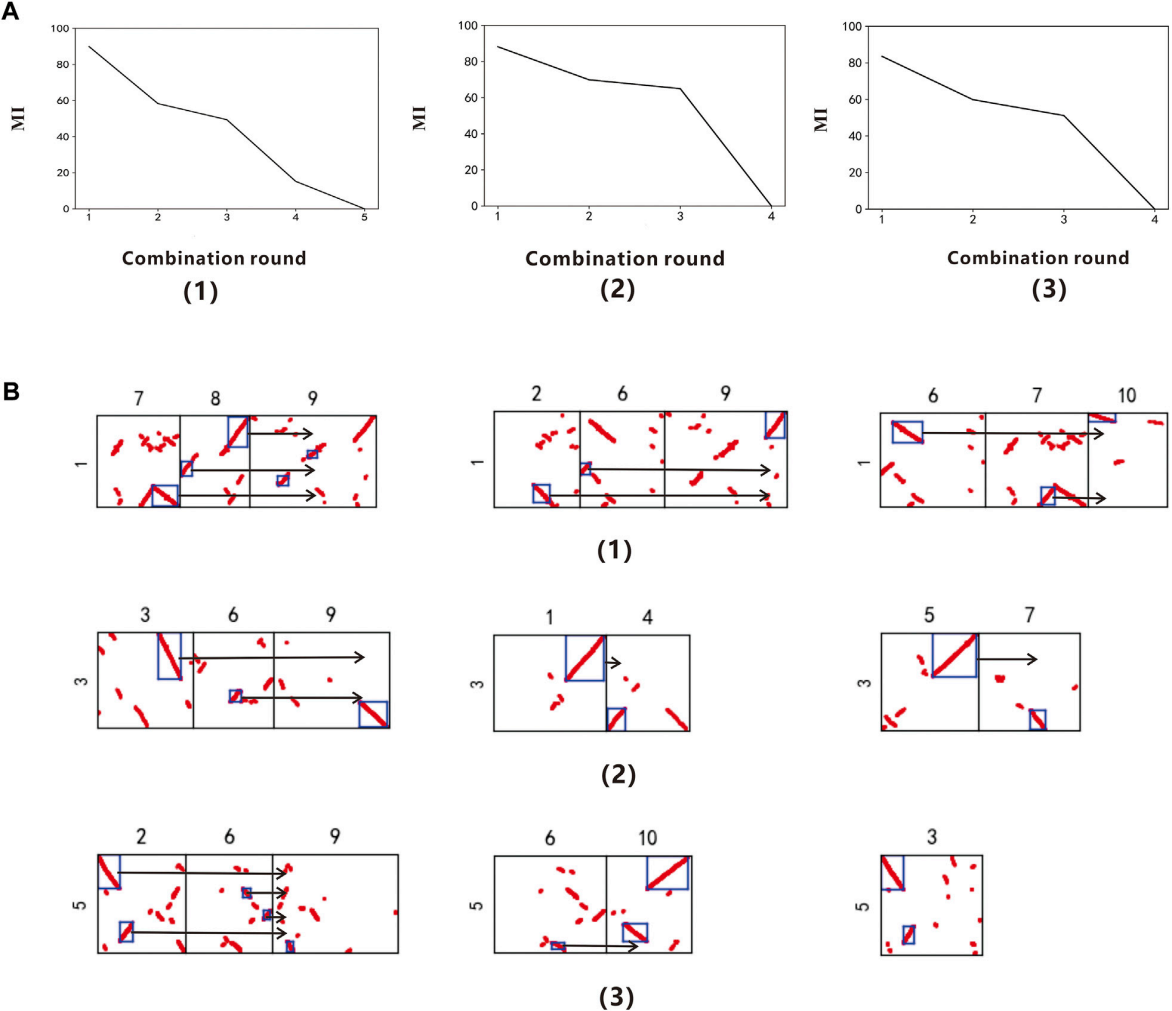
**(A)** Collinearity evaluation index line chart of *Arabidopsis thaliana* Chr.1, Chr.3 and Chr.5 **(B)** Combination figure of Brassica rapa polyploidy. **(A,B)** correspond to each other. (1) The *Arabidopsis thaliana* chromosome 1 (2) The *Arabidopsis thaliana* chromosome 3 (3) The *Arabidopsis thaliana* chromosome 5

In order to further verify the universality of the method, according to the procedure in 3.1, the model uses *Arabidopsis thaliana* as the reference genome to identify the polyploidy type of the target species *Brassica rapa*. Through reading the data of chromosomes 1, 3 and 5 of *Arabidopsis thaliana*, we finally get the gene collinearity evaluation index table of each chromosome in different combination rounds (Supplementary Table S2). In the collinearity evaluation index line chart (Figure 3A), it can be found that chromosomes 1, 3, and 5 of *Arabidopsis thaliana* had an obvious turning point when the combination round was 3. Therefore, it is determined that the *Brassica rapa*. had a whole genome triplication event recently. This conclusion can be found in Wang (2011a).

After determining the specific polyploidy, the result.csv file of *Arabidopsis thaliana* chromosomes 1, 3, and 5 is obtained, in which the data of chromosome 3 is shown in Table 4.

**TABLE 4:** *Arabidopsis thaliana* Chr. 3 result.csv file.

chr1	chr2	id	l_x	l_y	num	r_x	r_y	Sumy	Δy	Δx	Comro
3	3	1	2,919	5,436	857	4,001	2,763	4,793	2,673	1,082	1
9	3	1	3,322	1,458	827	4,386	8	4,793	1,450	1,064	1
6	3	1	1,571	2,132	260	2,021	1,462	4,793	670	450	1
1	3	1	2,581	5,431	1,036	3,948	2,863	3,799	2,568	1,367	2
4	3	1	3	1,231	501	631	0	3,799	1,231	628	2
5	3	1	1,955	5,432	1,374	3,668	3,021	3,533	2,411	1,713	3
7	3	1	1,555	1,130	373	1,998	8	3,533	1,122	443	3

According to the result.csv file, output the combined figure of *Brassica rapa* polyploidy (Figure 3B). When the combination round is 3, get the three groups with the highest scores, among which the chromosomes 7, 8, and 9 of the *Brassica rapa* can be combined into a relatively complete chromosome 1 of *Arabidopsis thaliana*, the corresponding MI is 89.88%; the chromosomes 2, 6, and 9 of the *Brassica rapa* can be combined into a relatively complete chromosome 1 of *Arabidopsis thaliana*, and the corresponding MI is 58.31%; the chromosomes 6, 7, and 10 of the *Brassica rapa* can be combined into a relatively complete chromosome 1 of *Arabidopsis thaliana*, and the corresponding MI is 49.38%. The chromosomes 3, 6, and 9 of the *Brassica rapa* can be combined into a relatively complete *Arabidopsis thaliana* chromosome 3 with MI of 88.16%; the chromosomes 1 and 4 of the *Brassica rapa* can be combined into a relatively complete *Arabidopsis thaliana* chromosome 3 with MI of 69.87%; the chromosomes 5 and 7 of the *Brassica rapa* can be combined into a relatively complete *Arabidopsis thaliana* chromosome 3 with MI of 64.98%. The chromosomes 2, 6, and 9 of the *Brassica rapa* can be combined into a relatively complete *Arabidopsis thaliana* chromosome 5, MI is 83.54%; the chromosomes 6 and 10 of the *Brassica rapa* can be combined into a relatively complete *Arabidopsis thaliana* chromosome 5, MI is 59.84%; the chromosomes 3 of the *Brassica rapa* can be combined into a relatively complete *Arabidopsis thaliana* chromosome 5, MI is 51.17%.

In Figure 3B, there are two seemingly identical collinearity fragments among the four fragments formed by *Arabidopsis thaliana* chromosome 3 and *Brassica rapa* chromosome 4, with the naked eye. But only one is labeled and used, because it has a small number of homologous genes. So this method can break through the limitations of the human eye, and find chromosome fragments with strong collinearity, as well as provide a basis for objective judgment of polyploidy.

## Discussion

The previous study has mostly used to observe the atlas with prior knowledge to identify the polyploid types of the species. This method has some major limitations, such as low efficiency, high dependence on prior knowledge, strong subjectivity, lack of objective evaluation criteria, and easy introduction of human error. In this paper, digital image processing technology was used to identify polyploid types based on clustering algorithms. The *K*_*S*_ dotplot of homologous genes was used as the research object, and the DBSCAN method was used to cluster. Then we can obtain the collinear fragments and automatically label collinear region. According to the gene collinearity evaluation index line chart of each combination, the model can determine the polyploid type and related chromosome combination. The study mainly focused on developing a polyploidization recognition algorithm and providing the method to speed up the evolutionary laws of gene structure associated with polyploidy research. PolyReco involves more than a simple labels of collinear regions, but also gives the polyploidy types through the collinearity evaluation index line chart and related chromosomes at the end. Compared with MCScanX (Wang et al., 2012), PolyReco labels the specific gene segments involved in the polyploidy events and improves the recognition efficiency of polyploidy. Compared to traditional methods, PolyReco reduces the dependence on prior knowledge, solves the limitations of the human eye in visual space, comply with artificial logic analysis and reasoning process. Moreover, the PolyReco can not only provides an effective method for large-scale rapid identification of genome polyploidy but also has important application value in distant hybrid breeding (Rabanus-Wallace et al., 2021).

In summary, the proposed PolyReco provides a reference model for processing automatically label collinear regions and recognize polyploidy. However, the *K*_*S*_ dotplot is sensitive to the size of the parameter Eps. When a large value is used for Eps, the fragmented collinearity segments are easy to cluster together. On the contrary, it is easy to separate continuous fragments so that complete collinearity fragments cannot be clustered. In the next step, we expect to study the DBSCAN clustering method based on adaptive Eps to further optimize the clustering effect.

## Data Availability

The original contributions presented in the study are included in the article/Supplementary Material, further inquiries can be directed to the corresponding author.
