# Supplementary material for: PolyReco: A Method to Automatically Label Collinear Regions and Recognize Polyploidy Events Based on the K S Dotplot
Source: Front Genet. 2022 Apr 20;13:842387. doi: 10.3389/fgene.2022.842387 (PMC9065682; doi:10.3389/fgene.2022.842387)
Supplement: Supplementary file 1 [file Table1.DOCX]

Supplementary Material

**Supplementary Table 1**|Evaluation index of gene collinearity of *Vitis vinifera* Chr.4, Chr.13 and Chr.14 in different combination rounds

|  | 1 | 2 | 3 | 4 |
| --- | --- | --- | --- | --- |
| Chr.4 | 67.87% | 42.19% | 8.14% | 0.00% |
| Chr.13 | 73.93% | 70.96% | 0.00% | Null |
| Chr.14 | 88.74% | 79.88% | 10.95% | 0.00% |
