# Supplementary material for: PolyReco: A Method to Automatically Label Collinear Regions and Recognize Polyploidy Events Based on the K S Dotplot
Source: Front Genet. 2022 Apr 20;13:842387. doi: 10.3389/fgene.2022.842387 (PMC9065682; doi:10.3389/fgene.2022.842387)
Supplement: Supplementary file 2 [file Table2.DOCX]

Supplementary Material

**Supplementary Table 2**| Evaluation index of gene collinearity of *Arabidopsis thaliana* Chr.1, Chr.3 and Chr.5 in different combination rounds

|  | 1 | 2 | 3 | 4 | 5 |
| --- | --- | --- | --- | --- | --- |
| Chr.1 | 89.88% | 58.31% | 49.38% | 15.22% | 0.00% |
| Chr.3 | 88.16% | 69.87% | 64.98% | 0.00% | Null |
| Chr.5 | 83.54% | 59.84% | 51.17% | 0.00% | Null |
